# An assessment of the quality of randomised controlled trials conducted in China

**DOI:** 10.1186/1745-6215-9-22

**Published:** 2008-04-24

**Authors:** Dalu Zhang, Peng Yin, Nick Freemantle, Rachel Jordan, Nanshan Zhong, KK Cheng

**Affiliations:** 1 Department of Public Health and Epidemiology, University of Birmingham, Edgbaston, Birmingham B15 2TT, UK; 2 Department of Primary Care and General Practice, Primary Care Clinical Sciences Building, University of Birmingham, Edgbaston, Birmingham B15 2TT, UK; 3 Guangzhou Institute of Respiratory Diseases, First Affiliated Hospital, Guangzhou Medical College, Guangzhou 510120, China

## Abstract

**Background:**

Despite the rapid increase in research in China, little is known about the quality of clinical trials conducted there.

**Methods:**

A systematic review and critical appraisal of randomised controlled trials (RCTs) conducted in China and published in 2004 was undertaken to describe their characteristics, assess the quality of their reporting, and where possible, the quality of their conduct. Randomised controlled trials in all disease areas and types of interventions, which took place in China and included Chinese citizens were identified using PubMed and hand searching the Journal Series of the Chinese Medical Association. Quality was assessed against a subset of criteria adapted from the CONSORT statement.

**Results:**

**Three hundred and seven **RCTs were included. One hundred and ninety-nine (64.8%) failed to report methods of randomization and 254 (82.4%) did not mention blinding of either participants or investigators. Reporting of baseline characteristics, primary outcome and length of follow-up was inadequate in a substantial proportion of studies. Fewer than 11% of RCTs mentioned ethical approval and only 18.0% adequately discussed informed consent. However, dropout rates were very favourable with nearly 44% of trials reporting a zero dropout rate.

**Conclusion:**

Reporting of RCTs in China requires substantial improvement to meet the targets of the CONSORT statement. The conduct of Chinese RCTs cannot be directly inferred from the standard of reporting; however without good reporting the methods of the trials cannot be clearly ascertained.

## Background

Randomised controlled trials (RCTs) are considered the 'gold standard' for assessing the effectiveness of pharmacological and other interventions in the field of medicine [1-4]. They are widely accepted as the best research design because they distribute both known and unknown prognostic factors between treatment groups by the play of chance [5-8] thereby minimizing the possibility that any treatment effect is due to bias or confounding, and providing the basis for valid statistical comparison [8].

However, RCTs vary in their methodological rigour, and it is well known that poor quality studies tend to produce systematically different results from larger, better quality studies, often erroneously showing larger treatment effects [9,10]. The conduct of studies cannot be assessed without clear reporting. Many medical journals now expect authors to adhere to internationally agreed standards of reporting thus allowing the reader to assess the conduct of each trial [11]; this has assisted in raising the standards of trial reporting in developed countries[12].

China is a developing country with the biggest population in the world. Research in China has been rapidly gaining momentum, but as yet there is no systematic evaluation of the current standard of trials conducted there. Evaluations of the quality of Chinese RCTs have been restricted to selected journals or fields, and often a limited list of quality indicators [13-19]. In one example, a recently published systematic review of the effectiveness of hyperbaric oxygen using Chinese RCTs found that the published papers reported inadequate information and were generally of poor quality [16].

We present a critical evaluation of randomised controlled trials conducted in China and published in 2004. Our aim was to describe their general characteristics, evaluate the quality of their reporting, and evaluate their conduct where adequately reported.

## Methods

The study was carried out according to a pre-defined protocol.

### Search strategy

Randomised controlled trials published in 2004 were identified through two broad sources:

1. Using the PubMed database. PubMed includes MEDLINE and OLDMEDLINE [20] but papers published in many non-English Journals are not listed. We searched PubMed for Chinese randomised controlled trials published in 2004 using the textwords 'chin*' and the PubMed filter for randomised controlled trials.

2. Since many of the main medical journals in China are not indexed in PubMed, or in any electronic database, we also accessed the online versions of each journal in the Journal Series of the Chinese Medical Association. The Journal Series of the Chinese Medical Association includes 71 journals, which comprise the main core medical journals in Mainland China and additionally the Chinese version of the British Medical Journal.

For both sources, reference lists of included studies were checked. No language or other limitations were imposed. Chinese text was translated into English by two authors fluent in Chinese (DZ and PY). Titles were initially scanned for relevance and abstracts read if titles were unclear. The full text of papers with no abstract was viewed and checked for eligibility.

### Inclusion and exclusion criteria

We included any papers reporting randomised controlled trials on all disease groups and all types of interventions, which were published in 2004, took place in China and included Chinese citizens. We excluded reports that did not include any participants from Mainland China. We excluded papers from Hong Kong and Taiwan where research and clinical practice are different from those in the Mainland.

### Assessment of quality

The CONSORT statement is an internationally agreed standard for reporting RCT[11]. It includes recommended items designed to report the methodology and conduct of a study that are common to many standard quality assessment checklists. We used or adapted a subset of the CONSORT indicators in order to assess both the quality of reporting and, in those studies where information was provided, the actual conduct of the study. We also added some customised indicators in order to extract basic descriptive information specific to the Chinese papers (Table 1). We did not use overall quality scores or categories to judge each paper because the use of summary scores could be problematic and often obscures individual aspects of quality [21].

**Table 1:** Indicators used to describe and evaluate included randomised controlled trials

**Indicator**		**Description**
**Descriptive indicators**		

1	Publication language	Chinese or English
2	Nationality of authors	Chinese, international or collaboration
3	Funding source	As reported
4	Disease area	Simple categories
5	Choice of comparator interventions	Placebo/alternative treatment/no treatment
6	Size of trial	Number of participants
7	Ethical committee approval	Yes/No
8	Informed consent from participants	As reported

**Quality of reporting: CONSORT indicators**		

9	Sample size	How was sample size determined?
10	Randomisation	Was the trial randomised?
11	Allocation concealment	What method was used to implement the random allocation sequence?
12	Blinding	Whether or not patients and/or investigators were blinded to group assignment
13	Baseline characteristics	Were the baseline demographic and clinical characteristics of each group reported
14	Primary outcomes	Did they report which outcome was designated as the primary outcome?
15	Length of follow-up	As reported
16	Loss to follow-up	As reported
17	Statistical reporting	Were confidence intervals or p values reported to indicate precision?

### Data extraction and analysis

One reviewer extracted data from all included papers. A second reviewer independently checked a random sample of 26% of the papers. Discrepancies were resolved where possible by discussion, and the sample results compared with the full results using Kappa scores. Data on the quality of the included papers were presented in tabular format accompanied by a critical description.

## Results

### Search results

**Figure 1:**
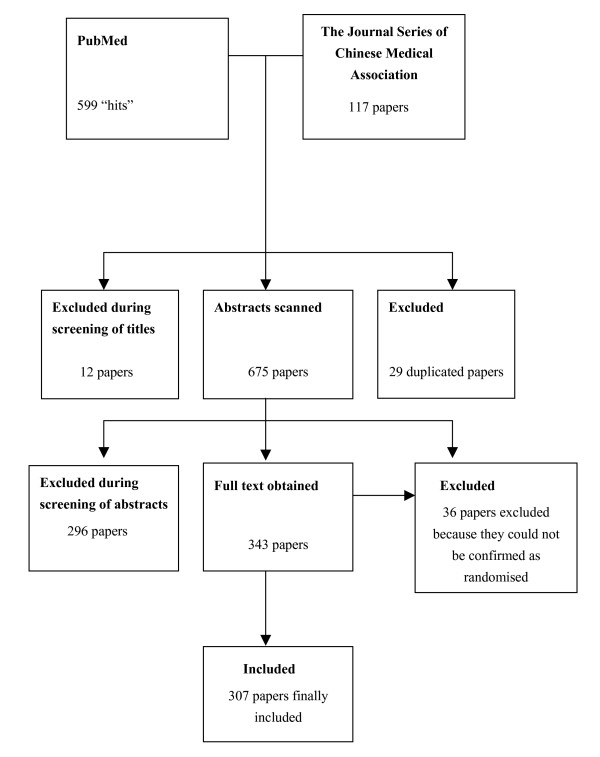
Flow chart of selection decisions.

Figure 1 describes the results of the search and the identification of eligible trials. Among 716 identified papers, 29 were initially excluded as they were duplicate publications of the same study. Only one paper was included for each study. Twelve were excluded on screening of the titles. Of the remaining 675 studies, 36 studies were excluded as they either were not, or could not be confirmed as, RCTs (Table 2), and 296 were excluded after reading the abstract. The full text of the remaining 343 papers was obtained, and finally 307 papers were included as confirmed RCTs. The full article list can be obtained from the contact author.

**Table 2:** Reasons for excluding papers

*Reason for exclusion*	*Number of papers*
Before/after studies	17
Brief report given only	6
Case control study	5
Phase II trial (no control arm)	4
Randomisation not mentioned in full text	2
Allocated by patients' choice	1
Allocated by patients' economic status	1

Total	36

### Agreement between reviewers

Agreement between the reviewers was good with a kappa score of greater than 0.7 for the main indicators (funding source, disease area, choice of comparator interventions, ethical committee approval, informed consent from participants, sample size, randomisation, allocation concealment, blinding, baseline characteristics, primary outcomes, loss to follow-up, length of follow-up, statistical reporting).

### Characteristics of the included trials

#### 1. Publication language

Of the 307 included RCT papers, 259 (84%) were written in Chinese. The remaining 48 papers were published in English.

#### 2. Nationality of authors

292 (95%) included papers were written by authors based in Chinese research institutes; the remaining papers were collaborations between Chinese and foreign researchers. There were no trials conducted or reported only by foreign researchers.

#### 3. Funding source

Of the 307 papers, 232 (75.6%) did not report their sources of funding. Funding was from provincial/municipal and national sources in 38 trials (12.4%) and 23 trials (7.2%) respectively. Foreign pharmaceutical companies, universities, international research agencies and the military financed five or fewer trials each.

#### 4. Disease area

Fifty (16.3%) of the RCTs focused on diseases of the digestive system (Table 3). The second most published disease area was disease of the circulatory system with 48 papers (15.6%), followed by tumours with 42 papers (13.7%) and diseases of the urogenital system (37 papers (12.1%)). Nervous system, motor system and respiratory system diseases each had approximately 5% share of the total number of trials as did the category of primary prevention or health promotion. One hundred and twenty-two of the included papers (39.7%) reported studies of traditional Chinese treatments such as traditional Chinese medicine (TCM), massage and acupuncture.

**Table 3:** Disease area of included trials

*Disease area*	*Number of papers*	*Percent*
Digestive system diseases	50	16.3
Circulatory system diseases	48	15.6
Tumours*	42	13.7
Urogenital system diseases	37	12.1
Nervous system diseases	16	5.2
Motor system diseases	16	5.2
Healthy population	15	4.9
Respiratory system diseases	15	4.9
Endocrine system diseases	12	3.9
Immune system diseases	9	2.9
Others	47	15.3

Total	307	100.0

*All types/locations of tumours

#### 5. Choice of comparator interventions

Thirty-nine (12.7%) of the included trials compared active treatment with a placebo group. Three of these were randomised controlled crossover trials where participants were blinded to the order of drug taken. In 179 (58.3%) trials the new treatment being tested was compared with an alternative named treatment, and in 79 (25.7%) trials the new treatment was compared with a treatment described as the "standard", but with no specific details. Seven additional studies included a control group receiving no treatment: three of them were health education and promotion projects, two of them were health rehabilitation and two drug trials. A further three papers described trials with three different treatment arms: active treatment, standard treatment and no treatment.

#### 6. Size of the trials

The number of participants in each included trial ranged from 3 to 19200, with a median of 78.

#### 7&8. Ethical issues (Ethics Committee Approval and Informed Consent)

Only 33 (10.8%) of the included Chinese trials reported approval by an ethics committee. The majority of the study reports (249 or 81%) did not provide any information about informed consent although 54 (17.6%) of papers stated that the participants did give consent. The remaining four studies stated that participants were included in the trial of their own free will.

### Quality of reporting

#### 9. Sample size

Only nine (2.9%) of the 307 papers mentioned sample size calculation.

#### 10&11. Methods of randomisation & allocation concealment

In nearly two-thirds of the included trials (Table 4) the authors failed to report details of their methods of randomisation. Seventy-three (23.8%) of the trials reported using a random number table to allocate participants; 13 (4.2%) a random allocation card; 11 (3.6%) a sealed envelope; 7 (2.3%) computer allocation and 4 (1.3%) the toss of a coin. Twenty-four trials allocated participants using visit order that were included in the "not clear" group. No trial mentioned allocation concealment.

**Table 4:** Methods of randomisation

*Method*	*Number of papers (%)*
Not clear	199 (64.8%)
Random number sheet	73 (23.8%)
Random allocation card	13 (4.2%)
Sealed envelope	11 (3.6%)
Computer allocation	7 (2.3%)
Toss of a coin	4 (1.3%)

Total	307 (100%)

#### 12. Blinding

254 (82.7%) papers provided no information about blinding of either participants or investigators. In 39 (12.7%) trials, both the investigators and participants were blinded. In 9 (2.9%) trials the participants were not blind, and in 5 (1.6%) the investigators were not blinded to the participants' treatments.

#### 13. Reporting of baseline characteristics

Eighty-nine (29%) of the included papers fully reported the baseline characteristics of the participants in a separate table. Two hundred and nine (67%) of the papers described baseline characteristics using either text or mixed tables, which also included results. In 9 papers (2.9%) only age was given in the baseline information, and in two papers no information was given other than a statement that the baseline characteristics matched in both arms.

#### 14. Reporting of primary outcomes

Only 11 (3.6%) of the included trials indicated which measure was used as the primary outcome; the remainder merely reported an ordered list of results from which it was not possible to distinguish which outcome was the primary.

#### 15. Length of follow-up

Table 5 details the distribution of length-of-follow-up for participants in the included studies. In 105 (34.2%) of papers, there was no information about the length of time for which participants were followed. The mean length of follow-up (where stated) was 166 days, although the median (interquartile range) was 56 (8–360) days.

**Table 5:** Length of follow up of included RCTs (days)

*Days of follow-up*	*Number of papers (%)*
Not clear	105 (34.2%)
0–30	85 (27.7%)
31–90	43 (14.0%)
91–365	43 (14.0%)
366–3650	31 (10.1%)

Total	307 (100.0%)

#### 16. Loss-to-follow-up

Over half of the trials (165 studies – 53.7%) reported that no participants had dropped out (Table 6). Sixty-four percent of all the clinical trials showed a drop out rate of 5% or less by the end of the study, and overall 70% of all the trials had a drop out rate lower than 10%. Fifty-seven (18.6%) studies failed to report dropout rates.

**Table 6:** Dropout rate of included trials

*Dropout rate (%)*	*Number of papers*	*Percent*	*Cumulative Percent*
Not clear	57	18.6	18.6
0	165	53.7	72.3
0.1–4.9%	27	8.8	81.1
5.0–9.9%	25	8.2	89.3
10.0–24.9%	20	6.5	95.8
>= 25%	13	4.2	100.0

Total	307	100.0	

#### 17. Statistical reporting

The majority of the papers (298 trials or 97.1%) conducted t-tests to examine the statistical significance of their results, and presented p values. In only 20 papers (6.5%) did the authors use confidence intervals to describe the uncertainty around their estimates.

## Discussion

### Key results

Study of trial quality is rare in developing countries, and tends to focus on limited clinical areas [22,23]. Although among Chinese publications there are a few paperswhich describe trial quality in specific journals or fields [13-19], this is the first systematic study to evaluate the quality of trial conduct and reporting in a sample which is likely to be more representative of Chinese RCTs in general.

Our review revealed that the standard of reporting of trials was generally poor, which concurs with the other published reports on Chinese trials [16-19]. For example, nearly two-thirds failed to report any information on their methods of randomisation, reinforcing previous work [18,19]. In the remainder there were various methods of random allocation, of which about a quarter reported using a computer-generated method or a random number table, which are the usually acceptable ways of randomisation. None of the trials discussed allocation concealment. If the allocation of the patient is not adequate and fully independent of the enrolling investigator, then this may allow either conscious or unconscious selection of participants into the trial, or into particular arms of the trial, thus introducing selection bias and undermining the randomisation. The internal validity of a randomised controlled trial has been shown to be directly associated with a clear description of appropriate methods of random allocation of participants, and concealment of their allocation [24].

Over 80% of trials provided no information about blinding of either participants or investigators. This confirms the result observed in a review of RCTs of traditional Chinese medicine[19]. Without blinding the groups may have been treated differently by the investigator and the outcomes not measured objectively, thus creating further assessment bias. Participants aware of their treatment may behave differently or have particular expectations [8], thus affecting the results.

Interestingly, among the included Chinese studies in this review, over half stated that none of their participants dropped out. This is unusual compared with trials in countries with more established research programmes, where a drop-out rate of below 5% is generally considered a very good result. Over 60% of the trials in this review reported a drop-out rate of less than 5%, and two-thirds less than 10%. The reasons behind these very low rates warrant further investigations.

The reporting of ethical issues was inadequate in the Chinese RCTs. Fewer than 11% of the trials reported having ethical committee approval, although the latter is a legal requirement in China [25]. Also, only a minority of the Chinese studies (17.4%) gave adequate details about informed consent procedures; a few mentioned that participants attended of "their own free will" but the remainder made no mention of consent. However, this level appears better than in a recent review of traditional Chinese medicine trials [19].

Compared with many published trials in developed countries[26], the standard of reporting in China is lagging behind, although there are still many fields in Western countries which have inadequate standards of reporting [27]. However, the application of the CONSORT statement has demonstrated benefits in improving reporting [28] and could be expected to do the same in China.

## Limitations

Although we undertook a thorough search for eligible studies using both PubMed and the Journal Series of the Chinese Medical Association, we may have missed relevant studies not included in the databases. The Journal Series of 71 Chinese journals comprises the core of the Chinese medical journals, but only approximately 20% of the total. RCTs which were not described as such in the abstract would have been excluded; however it is not clear how many such false negatives there would have been. Indeed failure to mention correctly the study design in the abstract is a mark of poor quality.

## Conclusion

Reporting of RCTs in China requires substantial improvement to meet the targets of the CONSORT statement. The conduct of Chinese RCTs cannot be directly inferred from the standard of reporting; however without good reporting the methods of the trials cannot be clearly ascertained. Research bodies in China should ensure that the reporting of RCTs is improved to meet internationally agreed standards, thereby allowing the conduct of their studies to be monitored and encouraging high quality standards.

## Competing interests

The authors declare that they have no competing interests.

## Authors' contributions

KKC initiated the project and oversaw the project. DZ contributed to the project design, management of searching, data extraction and analysis, and writing of the paper. PY reviewed 26% papers independently and conducted the statistical comparison between the reviewers. NF contributed to the project design, data analysis and interpretation. RJ commented and helped to draft the manuscript. NZ advised about the project and provided comments. All authors read and approved the final manuscript.
